# Analysis and Enhancement of Cluster Dither Angular Velocity Amplitude for Three-Axis Ring Laser Gyroscopes

**DOI:** 10.3390/s26216909

**Published:** 2026-10-31

**Authors:** Cheon Joong Kim, Jun Eon An, Haesung Yu, JunMin Park

**Affiliations:** 1 Agency for Defense Development, Daejeon 34022, Republic of Korea; kcj3651@gmail.com (C.J.K.); hae0817@add.re.kr (H.Y.); 2 Department of Electronics Engineering, Chungnam National University, Daejeon 34134, Republic of Korea

**Keywords:** angle random walk, ring laser gyroscope, cluster dither, angular velocity amplitude

## Abstract

In this paper, to achieve the required angular velocity amplitude of a cluster dither used with an RLG having an optical path of approximately 10 cm, an equation for the angular velocity amplitude of the cluster dither was derived and the design variables affecting the angular velocity amplitude were analyzed theoretically. The analysis indicated that increasing the angular velocity amplitude requires optimization of the driving-PZT attachment position, reduction in the effective end mass, and consideration of the equivalent Q-factor of the dither structure. Based on the theoretical analysis, a cluster dither was designed and fabricated, and test evaluations were performed to validate the proposed angular velocity amplitude prediction method. The cluster dither was fabricated from SUS304 and employed spokes with a uniform geometry. Validation of the proposed angular velocity amplitude analysis and design method through M&S and test evaluation yielded a mean angular velocity amplitude prediction error of 4.1% relative to measured values when the equivalent Q-factor determined from testing was applied.

## 1. Introduction

Backscattering of laser light from the mirrors inside the RLG (Ring Laser Gyroscope) resonator creates a region in which small input angular velocities cannot be measured. This region is referred to as the lock-in region. A representative method for eliminating the lock-in region is to apply rotational vibration to the RLG body [1,2,3]. A device that applies sinusoidal rotational vibration to the RLG body is called a dither, and various mechanical configurations are used [1].

**Figure 1:**
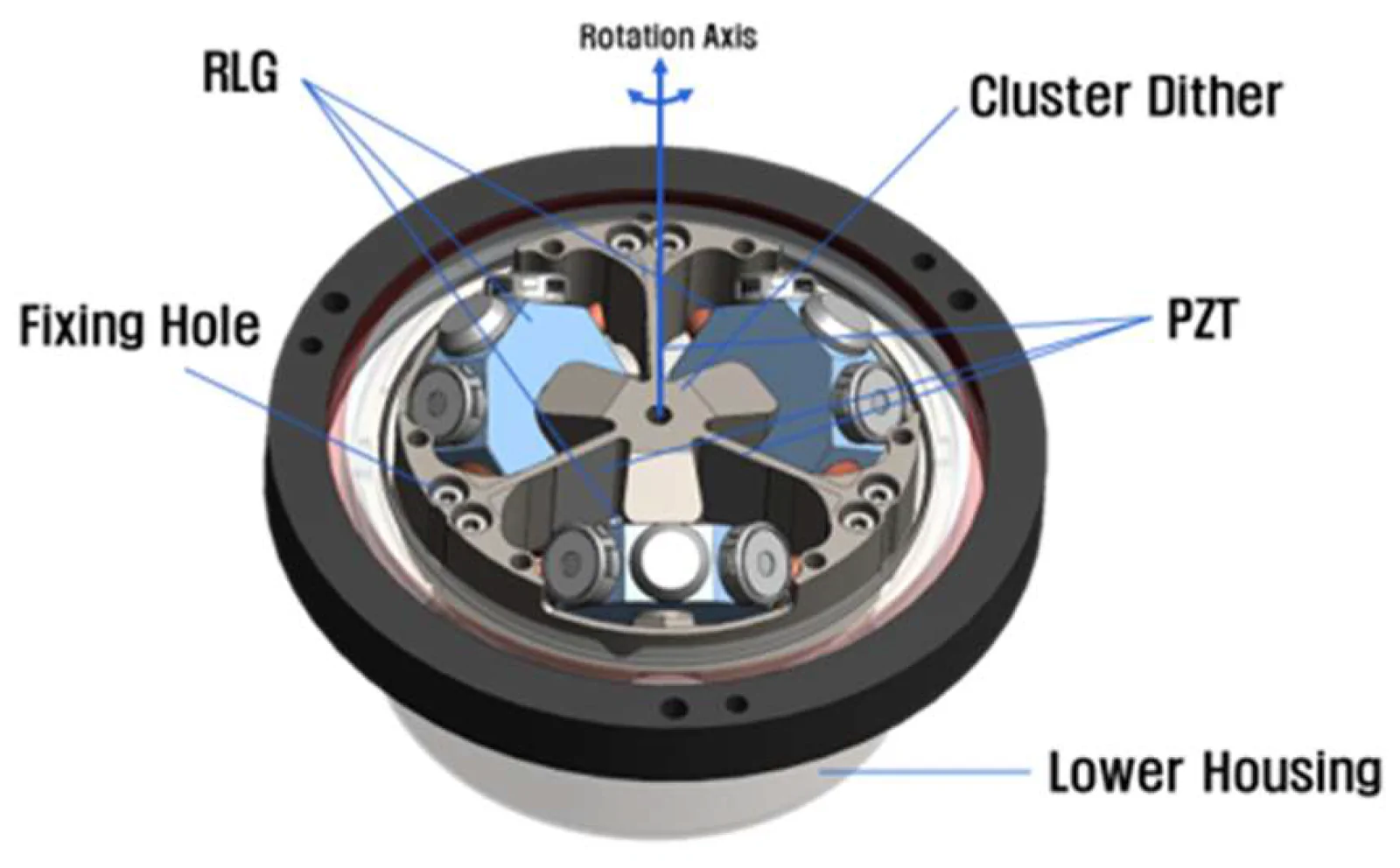
Lower part of an RLG-based inertial measurement unit with a cluster dither.

Generally, a dither is fixed at the center of the RLG body to generate sinusoidal vibration, and many studies have investigated its design and analysis [4,5,6,7,8]. However, as the gyro body is miniaturized, it becomes difficult to secure space for installing a dither at the center of the RLG. To address this problem, a cluster dither configuration capable of simultaneously applying sinusoidal vibration to three RLG bodies has been proposed [9]. Based on the cluster dither configuration proposed in Reference [9], a related study was recently conducted in Reference [10]. In the configuration of Reference [10], the rotation axis of the cluster dither is located at the center of the dither structure. The RLGs are arranged at 120° intervals about the rotation axis and mounted with an inclination relative to that axis. The configuration of the cluster dither applied to an RLG-based IMU is shown in Figure 1. The center of rotation of the cluster dither is located at the center of the dither, where the three RLG bodies are attached. One end of each dither spoke is fixed to the housing, whereas the other end is connected to the center of the dither, so that the three spokes mutually support one another. Therefore, unlike a single-axis dither, the cluster dither can be modeled as a clamped-supported cantilever beam. When a voltage with a frequency equal to the first-mode natural frequency of the dither is applied to the PZT (lead zirconate titanate) attached to the cluster dither, sinusoidal vibration can be generated.

Previous studies have presented a method for calculating maximum angular velocity based on energy equivalence [4,6] and a displacement analysis method based on static bending moments [5,7]. However, the equations proposed in those studies do not directly account for the clamped-supported cantilever boundary conditions of the three-axis cluster dither considered here or its steady-state resonance response including damping.

As summarized in Table 1, previous studies mainly addressed the calculation of maximum angular velocity using energy equivalence or static bending-displacement analysis. In contrast, the present study analyzes resonant angular velocity amplitude by applying sinusoidal PZT excitation and an equivalent Q-factor to a clamped-supported cantilever model of a three-axis cluster dither. The effects of PZT attachment position, effective end mass, and damping characteristics on angular velocity amplitude are investigated.

In this study, the angular velocity amplitude equation was derived by considering the dynamic response of the cluster dither at resonance under a sinusoidal bending moment generated by the PZT. The effects of PZT attachment position, effective end mass, and Q-factor were evaluated, and design methods for achieving the required angular velocity amplitude were proposed.

As the optical path length of an RLG decreases, the lock-in region tends to increase. Therefore, to minimize the time during which the RLG remains in the lock-in region, the dither angular velocity amplitude must be increased. Generally, an RLG with a 28 cm optical path is designed for a dither angular velocity amplitude of up to approximately 150 deg/s, whereas an RLG with a 6 cm optical path is designed for approximately 500–1000 deg/s [1,11,12,13,14]. The required dither angular velocity amplitude is determined by the size of the RLG lock-in region and the target angle random walk (ARW) performance [1,2,3]. Increasing the dither angular velocity amplitude is one design approach for reducing the lock-in effect and improving ARW performance. This may be achieved by raising the voltage applied to the PZT. However, this approach faces constraints such as increased power consumption and heat generation associated with high-voltage electronic circuits. Therefore, a dither design method that provides a larger angular velocity amplitude at the same applied voltage is required.

In this paper, an angular velocity amplitude equation is derived by assuming that a sinusoidal bending moment with a frequency equal to the natural frequency of the cluster dither is applied to the spoke. Using the derived angular velocity amplitude equation, the principal design variables affecting the dither angular velocity amplitude are identified, and design methods for increasing the angular velocity amplitude are proposed. In addition, the physical assumptions and applicability of the previous energy-equivalence and static-bending-moment methods were compared with those of the proposed resonance-response-based method, to examine the need to consider dynamic amplification in resonant angular velocity amplitude analysis. The validity of the proposed method is verified through angular velocity amplitude measurements of a fabricated cluster dither.

## 2. Angular Velocity Amplitude Requirements for the Cluster Dither

According to Equation (1), the ARW of the RLG is inversely proportional to the square root of the angular velocity amplitude of the cluster dither; therefore, increasing the dither angular velocity amplitude can improve the RLG ARW performance [1,2,3]. In Equation (1), $\Omega_{L}$ is the size of the lock-in region, $\Omega_{d}$ is the angular velocity amplitude of the cluster dither, and *S_cf_* is the scale factor [1,2,3]. However, because of the structural limitations of the cluster dither, the angular velocity amplitude cannot be increased indefinitely. Therefore, the required angular velocity amplitude must be determined by considering both the mechanical structure and performance of the cluster dither and the ARW performance requirement associated with the RLG optical path length. For the RLG with an optical path of approximately 10 cm shown in Figure 1, *S_cf_* is approximately 689 pulse/deg. If $\Omega_{L}$ is assumed to be approximately 0.1–0.125 deg/s and the target RLG ARW performance is set to 0.006 deg/√hr, the angular velocity amplitude required for the RLG is calculated from Equation (1) to be 200–300 deg/s. Here, 200–300 deg/s is the peak angular velocity actually applied to the RLG. Because the RLG is mounted at an inclination of approximately 54°, the cluster dither itself must generate an angular velocity amplitude of 340–510 deg/s, obtained by dividing the required RLG angular velocity amplitude by cos(54°). Therefore, considering design margin, 540 deg/s was set as the peak angular velocity amplitude of the cluster dither. This corresponds to a peak-to-peak angular velocity amplitude of 1080 deg/s. Here, the required value of 540 deg/s is the peak amplitude of the sinusoidal angular velocity of the cluster dither. As the peak-to-peak amplitude of a sinusoidal angular velocity is twice its peak amplitude, the design target corresponds to 1080 deg/s peak-to-peak. Whenever angular velocity amplitude is expressed as peak-to-peak in the following sections, this is explicitly indicated.

(1) $$RW=\Omega_{L}\sqrt{\frac{1}{2\pi \cdot Scf\cdot \Omega_{d}}}$$

## 3. Analysis and Enhancement of Cluster Dither Angular Velocity Amplitude

**Figure 2:**
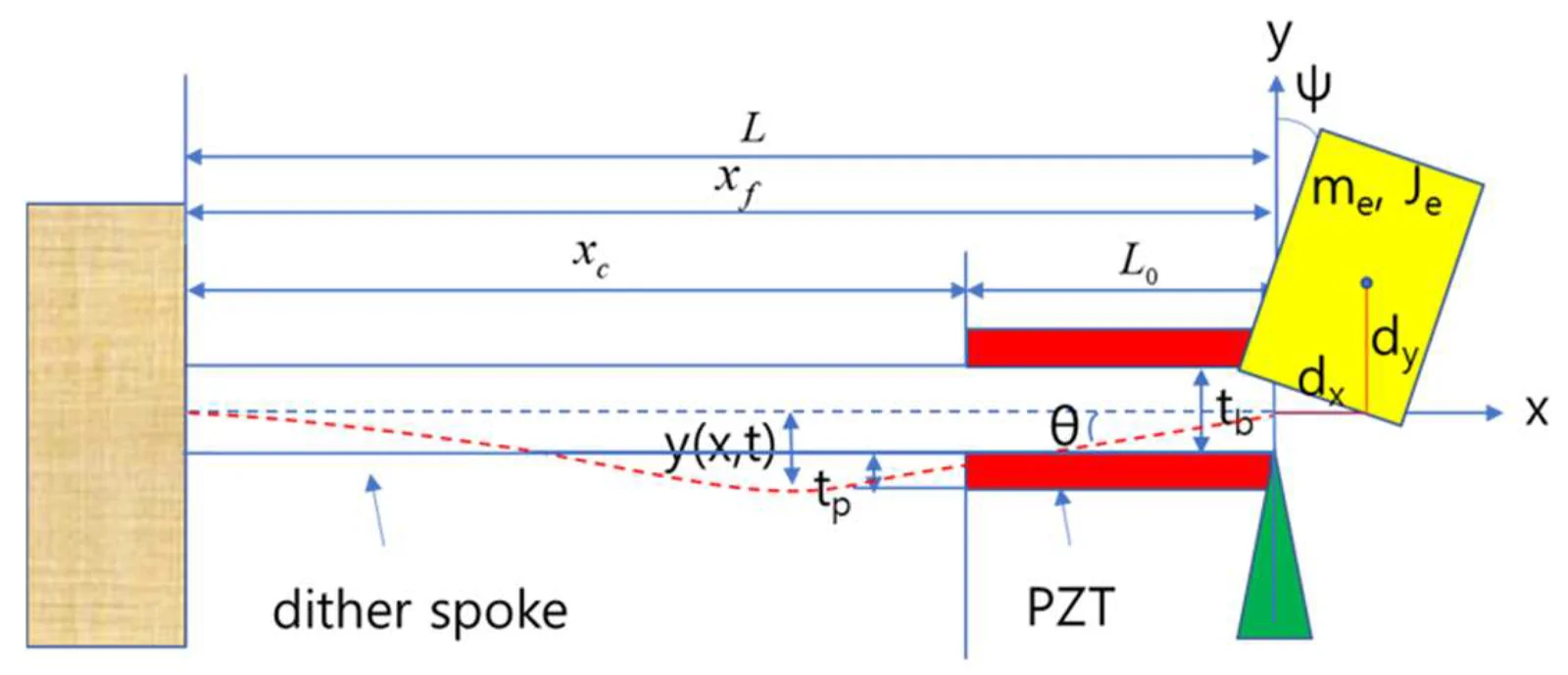
Configuration of a clamped-supported cantilever beam with an end mass.

This section derives the angular velocity amplitude equation of the cluster dither and proposes a method for increasing the angular velocity amplitude based on the derived equation. The cluster dither in Figure 1 simultaneously mounts three ring laser gyroscopes and has a structure in which the three dither spokes mutually support one another at the center of rotation. Therefore, it must be modeled as a clamped-supported cantilever beam, as shown in Figure 2.

Because the three spokes of the cluster dither mutually support one another at the central structure, the dither can be modeled as a clamped-supported cantilever, unlike the clamped-free cantilever of a conventional single-axis dither. This modeling approach was applied in the authors’ previous study [15] to the natural-frequency analysis of a cluster dither with partially tapered spokes, and its validity was examined through finite-element analysis and prototype testing. In the present study, this structural modeling approach is used to analyze the resonant angular velocity amplitude under PZT excitation for a uniform-cross-section spoke having the same cluster-dither support configuration.

**Figure 3:**
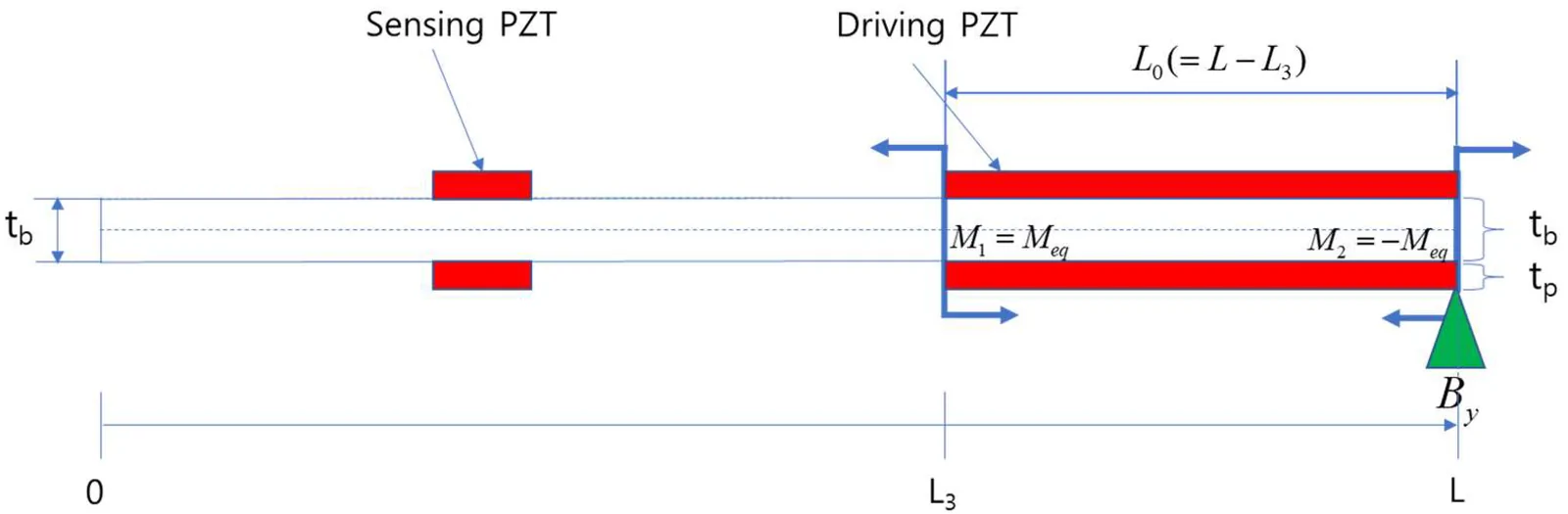
Configuration of a uniform dither spoke with PZT bending moments.

In Figure 2, the left side is the fixed end of the dither, and the yellow portion at the spoke end is the end attachment. The red portions attached to both sides of the spoke are the PZTs, and the triangle at the spoke end indicates that the end is supported. Figure 3 shows the dither spoke of Figure 2 separately. The concentrated moments $M_{1}$ and $M_{2}$ in Figure 3 are bending moments generated by the PZT. Some studies assume that these moments act at 1/2, the center of the driving PZT; however, in this paper, they are assumed to act at both ends of the PZT attachment region [5]. The PZT attached to the left side of the dither spoke in Figure 3 is the sensing PZT used to measure the dither angular velocity amplitude.

As shown in Figure 2, a uniform spoke cross-section was adopted in this study to validate the angular velocity amplitude analysis and enhancement method. Because the section properties of a uniform spoke are constant, it provides a suitable reference geometry for clearly examining the relationship between the first-mode shape function and angular velocity amplitude, and for evaluating major design variables such as PZT attachment position and effective end mass. The use of a uniform spoke in this study does not imply that it is optimal among all spoke geometries; comparisons and optimization involving profiled or partially tapered spokes are reserved for future work.

The equation of motion for transverse deflection of a general cantilever beam is given by Equation (2) [14,16]. In Equation (2), E is Young’s modulus of the dither spoke, I is the moment of inertia, ρ is the density, A is the cross-sectional area, and $p\left(x,t\right)$ is the externally applied force. Assuming that the displacement of the dither spoke varies with time and along the spoke length, the transverse displacement $y\left(x,t\right)$ can be expressed as Equation (3). In Equation (3), $Y_{r}\left(x\right)$ is the mode-shape function representing the deformation shape along the spoke length, $\eta_{r}\left(t\right)$ is the generalized coordinate representing the vibration amplitude of each mode as a function of time, and r denotes the mode order. Because each mode behaves as an independent oscillator, the total transverse displacement is expressed as the sum of all modes. To derive the dither angular velocity amplitude equation using Equations (2) and (3), both sides of Equation (2) are multiplied by the shape function $Y_{s}\left(x\right)$ of the sth mode and integrated over the entire dither spoke length. Rearranging the result into an ordinary differential equation gives Equation (4) [17]. The first integral term on the left-hand side of Equation (4) physically represents the effective stiffness, and expansion of the effective-stiffness integral gives Equation (5) [17]. Evaluation of Equation (5) requires the boundary conditions at the fixed and supported ends of the dither spoke.

(2) $$\frac{\partial^{2}}{\partial x^{2}}E(x)I(x)\frac{\partial^{2}y(x,t)}{\partial x^{2}}+\rho A\frac{\partial^{2}y(x,t)}{\partial t^{2}}=p(x,t)$$

(3) $$y(x,t)=\sum_{r=1}^{\infty }Y_{r}(x)\eta_{r}(t)$$

(4) $$\sum_{r=1}^{\infty }\eta_{r}(t)\int_{0}^{L}Y_{s}(x)\frac{d^{2}}{dx^{2}}E(x)I(x)\frac{d^{2}Y_{r}(x)}{dx^{2}}dx+\sum_{r=1}^{\infty }\frac{d^{2}\eta_{r}(t)}{dt^{2}}\int_{0}^{L}Y_{s}(x)\rho A(x)Y_{r}(x)dx=\int_{0}^{L}Y_{s}(x)p(x,t)dx$$

(5) $${\int_{0}^{L}Y_{s}(x)\frac{d^{2}}{dx^{2}}E(x)I(x)\frac{d^{2}Y_{r}(x)}{dx^{2}}dx=\left[\left(\frac{d}{dx}E(x)I(x)\frac{d^{2}Y_{r}(x)}{dx^{2}}Y_{s}(x)\right)-\left(E(x)I(x)\frac{d^{2}Y_{r}(x)}{dx^{2}}\right)\frac{dY_{s}(x)}{dx}\right]}_{0}^{L}+\int_{0}^{L}E(x)I(x)\frac{d^{2}Y_{s}(x)}{dx^{2}}\frac{d^{2}Y_{r}(x)}{dx^{2}}dx$$

The fixed-end boundary conditions of the cluster dither are the same as those of a general cantilever beam, whereas the end boundary conditions are different [18,19,20]. At the supported end of a clamped-supported cantilever beam, transverse displacement is constrained while rotation is free. Applying the supported-end conditions together with the fixed-end boundary conditions gives Equations (6)–(8). Equation (6) indicates that both the transverse displacement and slope are zero at the fixed end of the dither. Equation (7) indicates that the transverse displacement is zero at the end of the cluster dither. The end transverse displacement is zero because the three dither spokes mutually support one another at their ends. Equation (8) represents the equilibrium condition between the moment caused by the end attachment (the dither mounting part and ring laser gyroscope) and the bending moment at the end of the dither spoke, as shown in Figure 2. Because the transverse displacement at the end is constrained by the support, shear force acts as the support reaction. Therefore, the condition for the shear force $V\left(L\right)=0$ was not applied as an independent boundary condition. In contrast, because end rotation is free, the moment-equilibrium condition between the rotational inertia of the end attachment and the dither-spoke end moment was applied. As shown in Figure 2, in Equation (8), $J_{e}$ is the moment of inertia of the end attachment, $m_{e}$ is its mass, and d is the distance from the dither end to the center of mass of the end attachment. Because the center of mass of the end attachment is offset from the rotation axis by d, the inertial effect of the translational motion associated with rotation is included in the equivalent rotational inertia $J_{e}+m_{e}d^{2}$.

(6) $$y(0,t)=0,\frac{dy(0,t)}{dx}=0$$

(7) y(L,t)=0

(8) $$EI\frac{d^{2}y(L,t)}{dx^{2}}+(J_{e}+m_{e}d^{2})\frac{d^{3}y(L,t)}{dt^{2}dx}=0$$

Substituting the cluster dither boundary conditions in Equations (6)–(8) into Equation (5) and rearranging gives Equation (9), a second-order ordinary differential equation for $\eta_{r}\left(t\right)$. In Equation (9), $K_{r}$ is the effective stiffness of the dither spoke and $m_{r}$ is the effective mass, which are defined in Equations (10) and (11), respectively. In Equations (10) and (11), $\delta_{rs}$ is the Kronecker delta function. Dividing both sides of Equation (9) by $m_{r}$ and rearranging gives Equation (12). In Equation (12), $\omega_{r}$ is the rth natural frequency of the dither spoke, expressed in the standard form in which the natural frequency is calculated from stiffness and mass. Equation (12) is the undamped free-vibration equation. Adding the damping ratio ζ gives the standard second-order damped-system equation in Equation (13).

(9) $$m_{r}\frac{d^{2}\eta_{r}(t)}{dt^{2}}+K_{r}\eta_{r}(t)=\int_{0}^{L}Y_{r}(x)p(x,t)dx$$

(10) $$\int_{0}^{L}E(x)I(x)\frac{d^{2}Y_{s}(x)}{dx^{2}}\frac{d^{2}Y_{r}(x)}{dx^{2}}dx=K_{r}\delta_{rs}$$

(11) $$\int_{0}^{L}\rho A(x)Y_{s}(x)Y_{r}(x)dx+(J_{e}+m_{e}d^{2})\frac{dY_{s}(L)}{dx}\frac{dY_{r}(L)}{dx}=m_{r}\delta_{rs}$$

(12) $$\frac{d^{2}\eta_{r}(t)}{dt^{2}}+\frac{K_{r}}{m_{r}}\eta_{r}(t)=\frac{d^{2}\eta_{r}(t)}{dt^{2}}+\omega_{r}^{2}\eta_{r}(t)=\frac{1}{m_{r}}\int_{0}^{L}Y_{r}(x)p(x,t)dx$$

(13) $$\frac{d^{2}\eta_{r}(t)}{dt^{2}}+2\zeta \omega_{r}\frac{d\eta_{r}(t)}{dt}+\omega_{r}^{2}\eta_{r}(t)=\frac{1}{m_{r}}\int_{0}^{L}Y_{r}(x)p(x,t)dx$$

In Equation (13), $p\left(x,t\right)$ denotes the externally applied force. In the cluster dither, the external input can be expressed as the bending moment generated by the PZT attached to the dither spoke. The relationship between the PZT bending moment $M\left(x,t\right)$ and $p\left(x,t\right)$ is given by Equation (14) [21]. In Equation (14), $M\left(x,t\right)$ is modeled as follows. If the PZT is attached from $x_{c}$ to $x_{f}$ along the dither spoke and the bending moment $M_{eq}\left(t\right)$ is assumed to act at both ends of the PZT attachment region, Equation (15) is obtained [21]. In Equation (15), $H\left(x-x_{c}\right)$ and $H\left(x-x_{f}\right)$ are Heaviside functions. Substituting Equation (15) into Equation (14) and rearranging gives $p\left(x,t\right)$ as Equation (16). In Equation (16), $\delta \left(x-x_{c}\right)$ and $\delta \left(x-x_{f}\right)$ are Dirac-delta functions. Finally, substituting Equation (16) into the right-hand side of Equation (13) and rearranging gives Equation (17).

(14) $$p(x,t)=\frac{\partial^{2}M(x,t)}{\partial x^{2}}$$

(15) $$M(x,t)=M_{eq}(t)(H(x-x_{c})-H(x-x_{f}))$$

(16) $$p(x,t)=M_{eq}(t)\frac{\partial \delta (x-x_{c})-\partial \delta (x-x_{f})}{\partial x}=M_{eq}(t)(\delta^{\prime }(x-x_{c})-\delta^{\prime }(x-x_{f}))$$

(17) $$\frac{1}{m_{r}}\int_{0}^{L}Y_{r}(x)M_{eq}(t)(\delta^{\prime }(x-x_{c})-\delta^{\prime }(x-x_{f}))dx=\frac{M_{eq}(t)(Y_{r}^{\prime }(x_{c})-Y_{r}^{\prime }(x_{f}))}{m_{r}}$$

As described above, when a voltage is applied to the PZT attached to the dither spoke to generate a sinusoidal bending moment at a frequency equal to the dither natural frequency $\omega_{r}$, the dither undergoes sinusoidal motion with a constant steady-state response amplitude. To model the sinusoidal bending moment, $M_{eq}\left(t\right)$ in Equation (17) is expressed as the sinusoidal function in Equation (18). In Equation (18), $M_{0}$ denotes the amplitude of the bending moment generated by the voltage applied to the PZT. Substituting Equation (18) into Equation (17) and then applying Equation (17) to Equation (13) to obtain the steady-state response of $\eta_{r}\left(t\right)$ gives Equation (19).

(18) $$M_{eq}(t)=M_{0}\sin(\omega_{r}t)$$

(19) $$\eta_{r}(t)=\frac{1}{m_{r}}\frac{M_{0}(Y_{r}^{\prime }(x_{c})-Y_{r}^{\prime }(x_{f}))}{2\zeta \omega_{r}^{2}}\sin(\omega_{r}t-\psi )$$

Substituting Equation (19) into Equation (3) and rearranging gives the transverse displacement of the dither in Equation (20). To obtain the angular velocity amplitude of the dither, Equation (20) is differentiated once with respect to the dither-spoke length and then once with respect to time, yielding the angular velocity expression in Equation (21). Therefore, Equation (21) is the final cluster dither angular velocity amplitude equation used in this study.

(20) $$y(x,t)=\sum_{r=1}^{\infty }Y_{r}(x)\frac{1}{m_{r}}\frac{M_{0}(Y_{r}^{\prime }(x_{c})-Y_{r}^{\prime }(x_{f}))}{2\zeta \omega_{r}^{2}}\sin(\omega_{r}t-\psi )$$

(21) $$\dot{\theta }(x,t)=\sum_{r=1}^{\infty }\frac{1}{m_{r}}\frac{M_{0}Y_{r}^{\prime }(x)(Y_{r}^{\prime }(x_{c})-Y_{r}^{\prime }(x_{f}))}{2\zeta \omega_{r}}\cos(\omega_{r}t-\psi )$$

According to Equation (21), the dither angular velocity amplitude is inversely proportional to the effective mass and damping ratio, and is proportional to the PZT bending moment $M_{0}$, the slope of the shape function, and the difference between the shape-function slopes at the start and end points of the PZT attachment. From the mathematical form of Equation (21) alone, the natural frequency appears to be inversely proportional to the angular velocity amplitude. However, the natural frequency itself is a function of the effective stiffness and effective mass of the dither spoke, and these two quantities are coupled to several design variables. Therefore, the relationship between the natural frequency and angular velocity amplitude can also change as the design variables change. Thus, even though a natural-frequency term appears in the denominator of Equation (21), there are limitations to interpreting an increase or decrease in the angular velocity amplitude by treating only the change in natural frequency as an independent variable.

As can be seen from Equations (19) and (20), the shape function $Y_{r}\left(x\right)$ is required to calculate the effective mass accurately, and according to Equation (21), the dither angular velocity amplitude is also affected by the first-mode shape function. For a uniform cantilever beam, the general shape function $Y_{r}\left(x\right)$ is expressed as a combination of sin/cos and sinh/cosh functions [14,16,22]. However, to simplify the calculation, the first-mode shape function can be represented by a high-order polynomial trial/admissible shape function satisfying the boundary conditions [23]. In this paper, the fourth-order polynomial first-mode approximate shape function in Equation (22) was used so as to satisfy the boundary conditions in Equations (6)–(8). In Equation (22), A is the amplitude of the shape function, and L is the dither-spoke length shown in Figure 2 and Figure 3. Substituting Equation (22) into the boundary conditions in Equations (6)–(8) and rearranging determines the coefficients of Equation (22).

Many studies have investigated the bending moment $M_{0}$ generated when voltage is applied to the PZTs attached to both sides of a dither spoke [5,21,24,25,26]. In this paper, the bending moment $M_{0}$, derived by accounting for the effects of PZT stiffness and thickness on the stiffness of the dither spoke, is used [21,25]. The PZT bending moment $M_{0}$ used in this study is expressed as Equation (24). In Equation (24), $E_{p}$ is the Young’s modulus of the PZT, $d_{31}$ is the piezoelectric constant of the PZT, $h_{b}$ is the depth of the dither spoke, V is the magnitude of the applied voltage, and $t_{b}$ and $t_{b}$ denote the thicknesses of the PZT and dither spoke, respectively.

According to Equation (21), the angular velocity is proportional to the difference between the shape-function slopes at the start and end points of the PZT attached to the dither spoke. In Equation (21), $x_{f}$ is assumed to be L, and the function composed of the shape-function slopes is defined as Equation (23). To increase the dither angular velocity amplitude, the PZT attachment start point $x_{c}$ must be selected so that Equation (23) is maximized. Differentiating Equation (23) once and finding the point at which the derivative is zero gives Equation (25), which determines the optimum PZT attachment start point based on the first-mode shape function and shows that the dither angular velocity amplitude can be increased by changing the PZT attachment position. In this paper, the PZT attachment end point $x_{f}$ was set to *L*, the end of the cluster dither.

Using the cluster dither angular velocity amplitude equation derived above, Equation (21), the final angular velocity amplitude generated when a sinusoidal voltage at the natural frequency of the dither is applied to the PZT can be written as Equation (26). The first-mode effective mass $m_{1}$ in Equation (26) can be calculated by substituting Equation (22) into Equation (11), and the bending moment $M_{0}$ can be calculated using Equation (24). The first derivative of the shape function in Equation (26) can be calculated by differentiating Equation (22) once with respect to *x* and substituting the corresponding position values.

The effects of the dither design variables on angular velocity amplitude, as obtained from Equation (26), are summarized in Table 2. For design variables 1–5 in Table 2, their direct effects on angular velocity amplitude can be identified from Equation (26). Variables 6–18 are related to the dither natural frequency and effective mass and were added for a more detailed analysis of their effects. The influence of each variable can be examined using Equations (10), (11) and (26).

(22) $$Y_{1}(x)=A\left(a+b(\frac{x}{L})+c{\left(\frac{x}{L}\right)}^{2}+d{\left(\frac{x}{L}\right)}^{3}+e{\left(\frac{x}{L}\right)}^{4}\right)$$

(23) $$g(x_{c})=Y_{1}^{\prime }(L)(Y_{1}^{\prime }(x_{c})-Y_{1}^{\prime }(L))$$

(24) $$M_{0}=\left(\frac{\frac{E_{b}t_{b}}{E_{p}t_{p}}}{6+\frac{E_{b}t_{b}}{E_{p}t_{p}}}\right)E_{p}d_{31}h_{b}V_{pp}(t_{b}+t_{p})$$

(25) $$x_{c}=L\left(\frac{-3d\pm \sqrt{9d^{2}-24ce}}{12e}\right)$$

(26) $$\Omega_{1}=\frac{M_{0}Y_{1}^{\prime }(L)(Y_{1}^{\prime }(x_{c})-Y_{1}^{\prime }(x_{f}))}{2\zeta m_{1}\omega_{1}}$$

In Equation (26), the damping ratio ζ varies with the dither material and geometry and has a characteristic proportionality to the loss factor of the dither material. The damping characteristics of the cluster dither can be evaluated through a resonance-response sweep test. In such a test, let $f_{p}$ denote the resonance frequency at which the angular velocity amplitude is maximum; for the maximum angular velocity amplitude at $f_{p}$, let $f_{L}$ and $f_{R}$ denote the frequencies on the left and right sides, respectively, at which the angular velocity amplitude becomes $\frac{1}{\sqrt{2}}$. The overall measured average Q-factor $\frac{f_{P}}{f_{R}-f_{L}}$ can then be obtained. If the overall measured average Q-factor represents the actual damping characteristics of the system, it can be used as the equivalent Q-factor $Q_{eq}$. However, in an actual resonance-response sweep test, the slopes of the angular velocity amplitude on the left and right sides of the first-mode resonance frequency can be strongly asymmetric. In this case, the overall measured average Q-factor may not adequately represent the actual damping characteristics of the system; therefore, an equivalent Q-factor suitable for angular velocity amplitude prediction must be selected separately. According to the Modal Strain Energy Method, an equivalent modal Q-factor can be evaluated by taking a strain-energy-weighted average of the inverse local Q-factor, that is, the loss factor, at each location. Therefore, to represent spatially nonuniform damping characteristics by a single equivalent parameter, an energy-based equivalent Q-factor can be defined as Equation (27) [27,28,29]. Equation (27) shows that the equivalent Q-factor can be determined by the material and geometry of the dither spoke. In Equation (27), $Q\left(x\right)$ denotes the Q-factor along the dither-spoke length. Using the relationship $\zeta =1/2Q_{eq}$ between the equivalent Q-factor and the damping ratio to obtain the damping ratio, and substituting $\zeta =1/2Q_{eq}$ into Equation (26) and rearranging it as a function of $Q_{eq}$, gives Equation (28). Reference [4] reported an equivalent Q-factor of 150 from a Q-factor measurement test on a single-axis dither. In this study as well, the equivalent Q-factor of the cluster dither should preferably be measured first for accurate angular velocity amplitude analysis. However, for analytical convenience at the initial design stage, the value reported in Reference [4] was used as a basis, and considering that the cluster dither spokes in this study are longer than those in Reference [4], an equivalent Q-factor of 100 was assumed for the initial angular velocity amplitude prediction.

(27) $$Q_{eq}=\frac{\int_{0}^{L}EI(x){\left(Y_{1}^{\prime \prime }(x)\right)}^{2}dx}{\int_{0}^{L}\frac{EI(x){\left(Y_{1}^{\prime \prime }(x)\right)}^{2}}{Q(x)}dx}$$

(28) $$\Omega_{1}=\frac{M_{0}Q_{eq}Y_{1}^{\prime }(L)(Y_{1}^{\prime }(x_{c})-Y_{1}^{\prime }(x_{f}))}{m_{1}\omega_{1}}$$

Equation (27) theoretically defines the equivalent Q-factor when the damping characteristics are spatially nonuniform. However, because the distribution of the local Q-factor along the spoke length was not measured directly in this study, the equivalent Q-factor was not calculated directly using Equation (27). Instead, after substituting several candidate Q-factors obtained from the resonance search test into Equation (28), the predicted angular velocity amplitudes were compared with the experimental results to select the equivalent Q-factor that best represents the damping characteristics of the resonance response.

Each relationship in Table 2 represents the direct mathematical dependence between the corresponding variable and the dither angular velocity amplitude while the effects of the other design variables are held constant. Therefore, when structural changes simultaneously alter the natural frequency and effective mass, the actual effect on angular velocity amplitude can differ from the simple relationships in Table 2.

## 4. M&S Results for the Analysis and Enhancement of Cluster Dither Angular Velocity Amplitude

**Figure 4:**
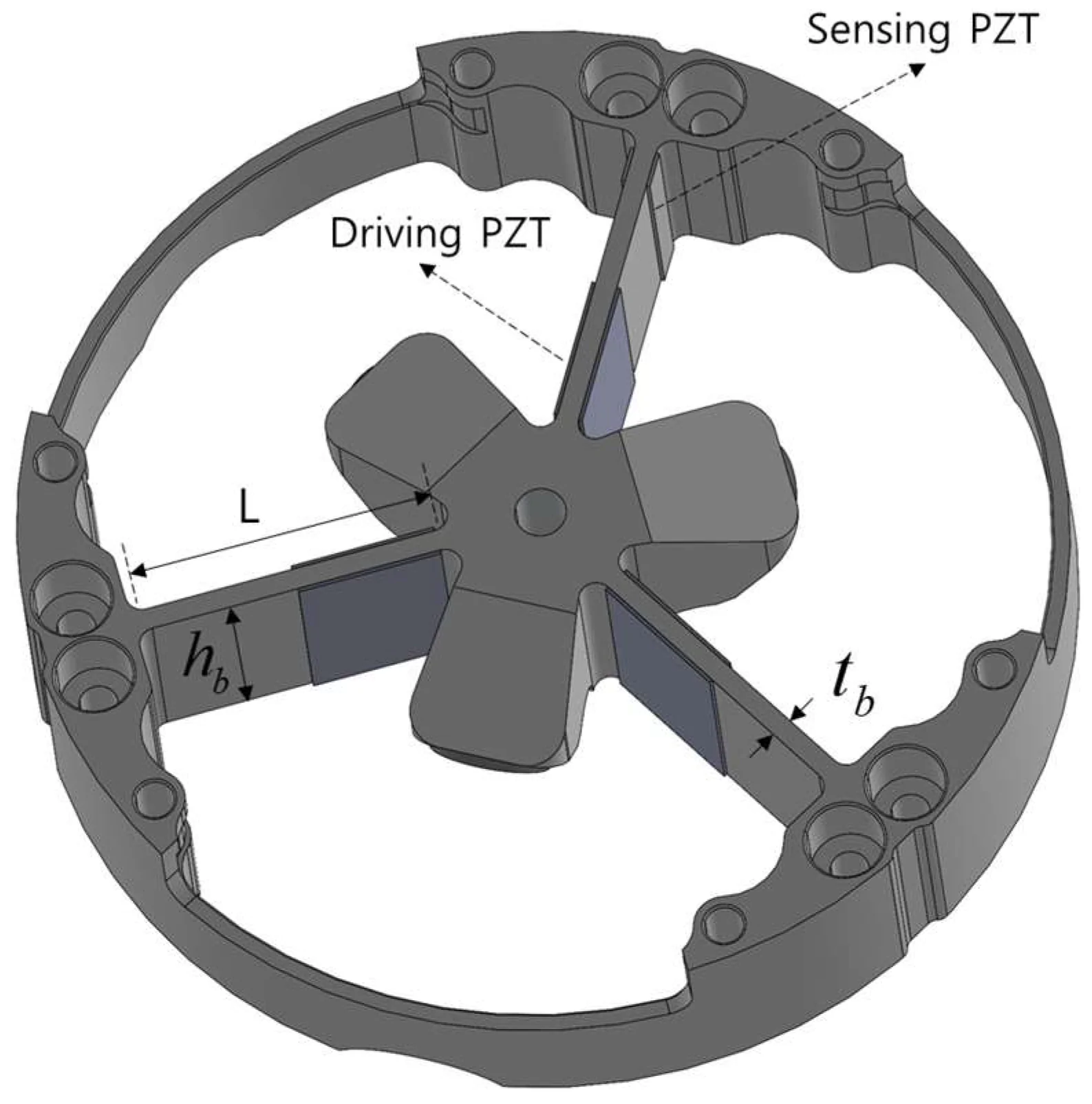
Geometry of the designed and fabricated cluster dither.

Based on Equation (28) and the angular velocity amplitude equation and influence analysis in Table 2, the cluster dither was designed to increase its angular velocity amplitude. First, because the effective mass is inversely proportional to the dither angular velocity amplitude, the thickness of the ring laser gyroscope was reduced and the geometry of the dither mounting part was modified to reduce the mass and moment of inertia of the end attachment. The geometry of the designed cluster dither is shown in Figure 4. The dither-spoke depth $h_{b}$ in Figure 4 was set to the maximum allowable value of 19 mm so as to maximize the value in Equation (24), the PZT bending-moment equation. Because the outermost diameter of the dither had already been determined, the dither-spoke length L was set to 24.24 mm by considering the size of the dither fixing region.

**Figure 5:**
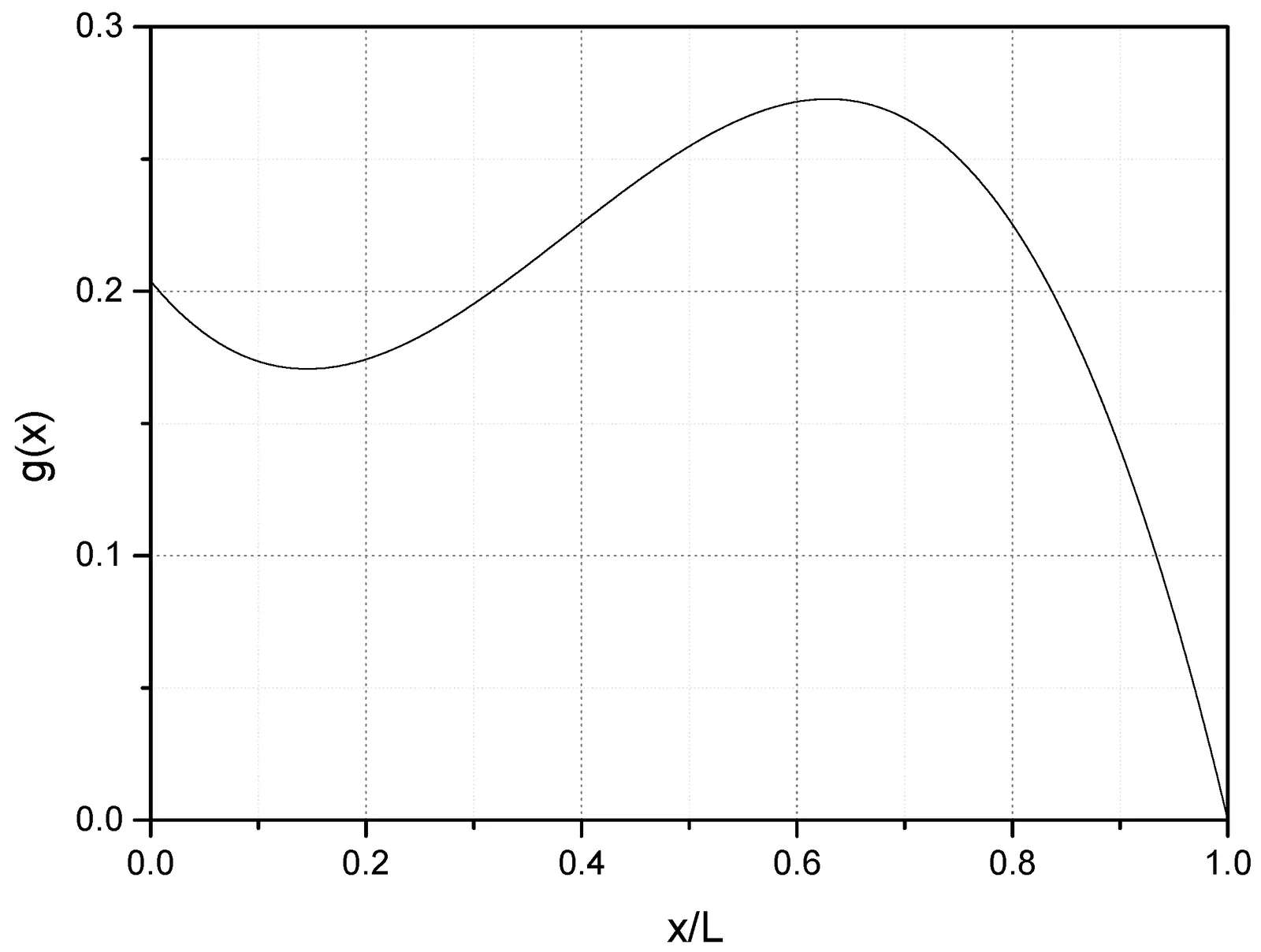
g(x) as a function of dither-spoke length.

Except for the already determined dither-spoke length and depth, the principal design variables related to increasing angular velocity amplitude are the material Q-factor, Young’s modulus and density, the dither-spoke thickness, and the PZT size and attachment position. SUS304 was selected as the cluster dither material because it is readily available, inexpensive, and has a high Young’s modulus. In addition, the optimum PZT attachment start point $x_{c}$ was determined based on the first-mode shape function so as to maximize the dither angular velocity amplitude. Substituting the design values in Table 3 into Equation (25) and calculating the position at which the difference in shape-function slopes is maximum showed that the maximum occurs when $x_{c}$ is 0.6*L*. To identify the optimum PZT attachment position more clearly, the M&S results obtained using Equation (23) are plotted as a function of dither-spoke length in Figure 5. Figure 5 confirms that the PZT attachment start point for increasing the angular velocity amplitude of the cluster dither is approximately 0.6L. The principal dimensions and variables of the designed cluster dither are summarized in Table 3.

**Figure 6:**
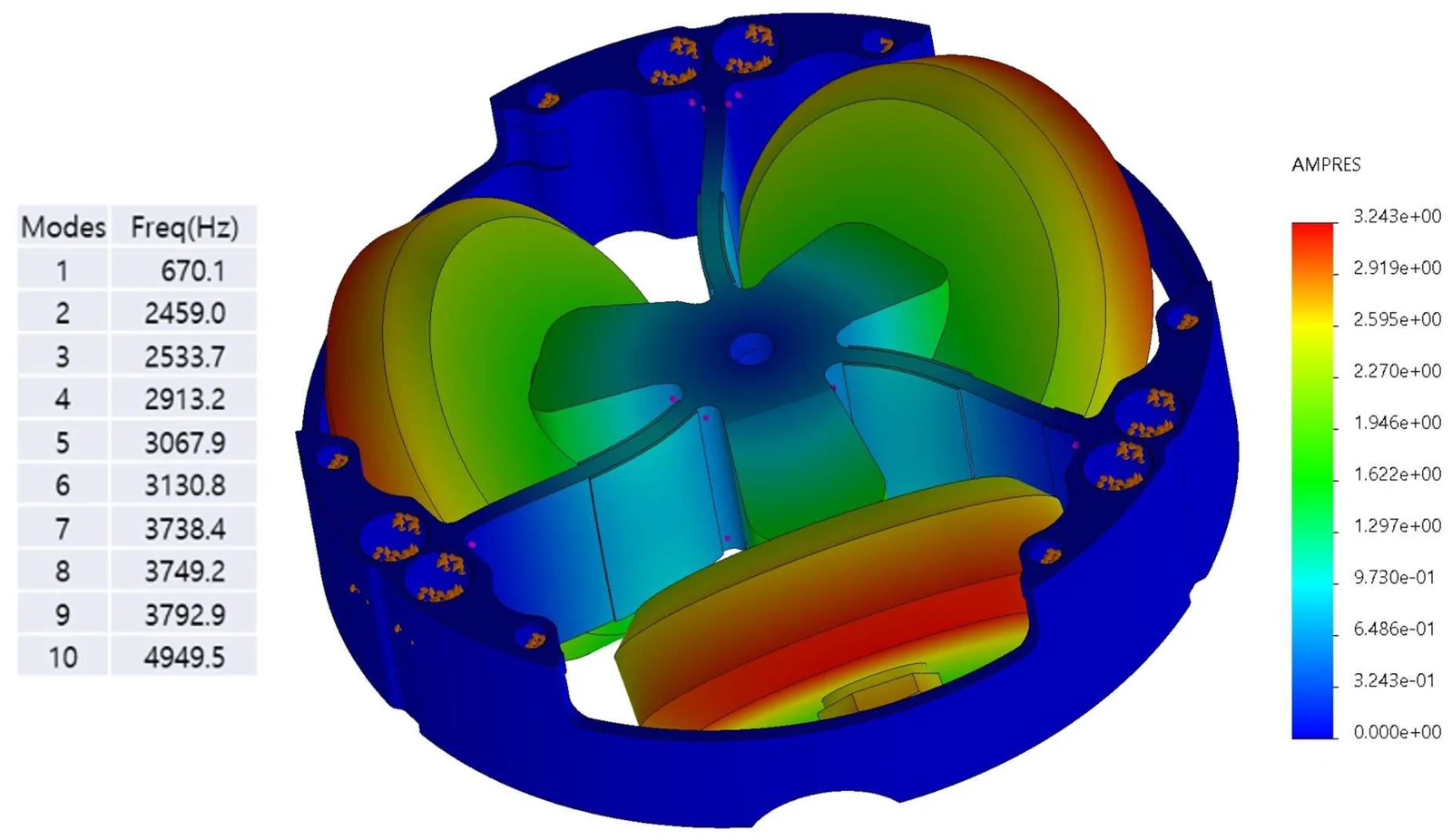
Modal analysis of the cluster dither.

In Table 3, $L_{0}$ denotes the width of the PZT shown in Figure 2. To examine the structural validity of the adopted clamped-supported cantilever model, finite-element modal analysis was performed using SOLIDWORKS 2022, a commercial engineering program, for a cluster dither with uniform-section spokes. The model included the three spokes, central structure, and end attachment, with fixed boundary conditions at the outer fastening portions and with the mass and inertial effects of the central structure and attachments considered. Figure 6 shows the first mode shape obtained from the finite-element analysis. The calculated first-mode natural frequency was 670.1 Hz; this result was compared with the prototype resonance frequencies measured in Section 5 to assess model validity. These results supported examination of the main vibration characteristics and applicability of the clamped-supported model for angular velocity amplitude analysis. However, assembly tolerances and structural asymmetry were not evaluated separately; the model’s application is therefore limited to the structure and first resonance mode investigated here.

The PZT Young’s modulus $E_{p}$ and piezoelectric constant $d_{31}$ required to calculate the PZT bending moment using Equation (24) were set to 61 GPa and 210 × 10^−12^, respectively. These values were selected with reference to the material properties of the PIC151 PZT model used to fabricate the actual cluster dither [30]. The PZT applied voltages were assumed to be 40, 80, and 120 $V_{pp}$.

The angular velocity amplitude predictions obtained by substituting the design values in Table 3 into Equation (28) and assuming PZT applied voltages of 40, 80, and 120 $V_{pp}$ are listed in Table 4. According to Table 4, an applied voltage of at least approximately 68 $V_{pp}$ is required to satisfy the peak-to-peak angular velocity amplitude requirement of 1080 deg/s presented in Section 2. However, this required voltage can vary depending on the measurement-based equivalent Q-factor appropriate for predicting the angular velocity amplitude of each dither model. In the initial prediction, in which the equivalent Q-factor was assumed to be Q = 100, an applied voltage of at least approximately 68 $V_{pp}$ was required.

## 5. Validation of the Cluster Dither Angular Velocity Amplitude Analysis and Enhancement Method

**Figure 7:**
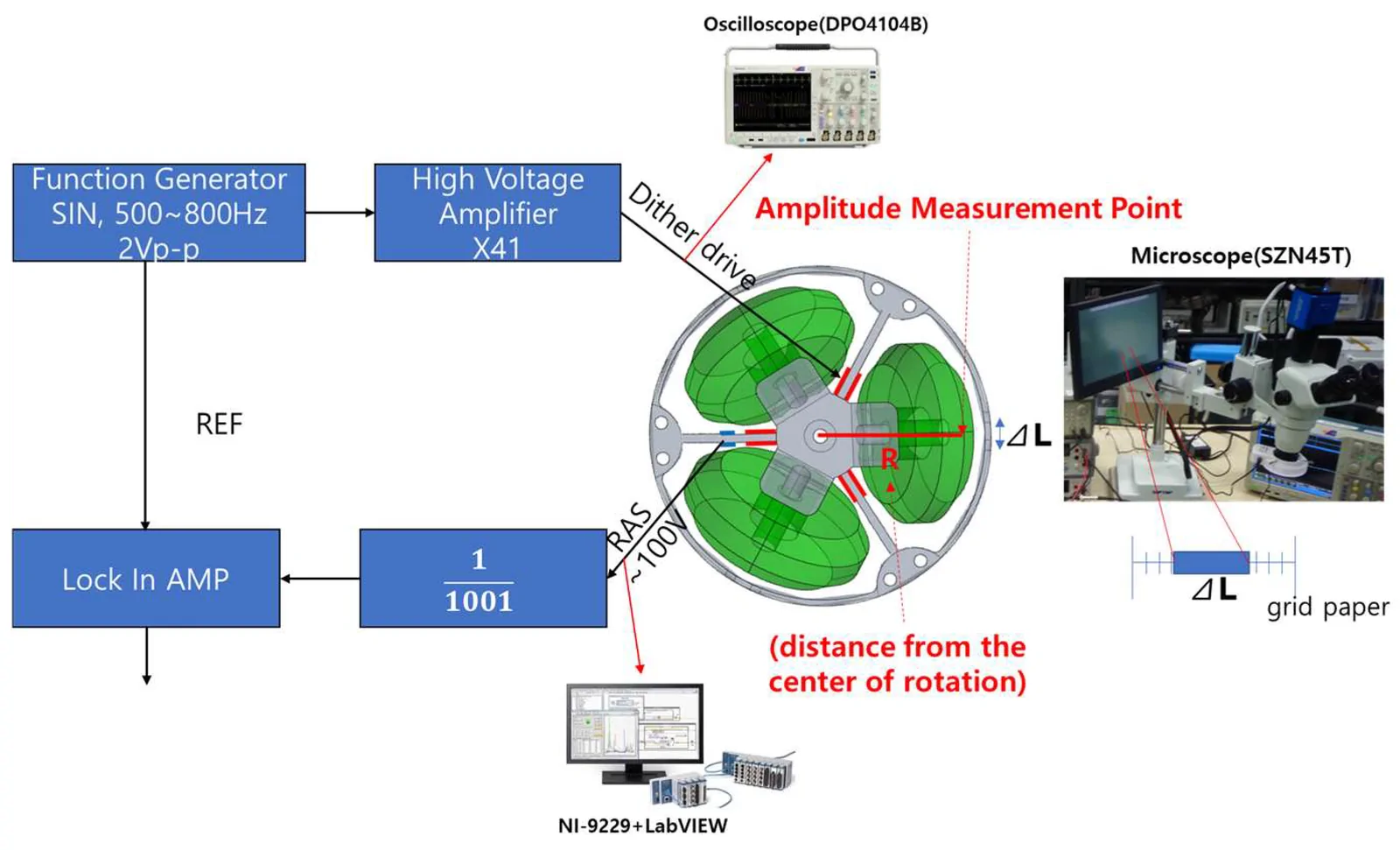
Test setup for measuring the angular velocity amplitude of the fabricated cluster dither.

**Figure 8:**
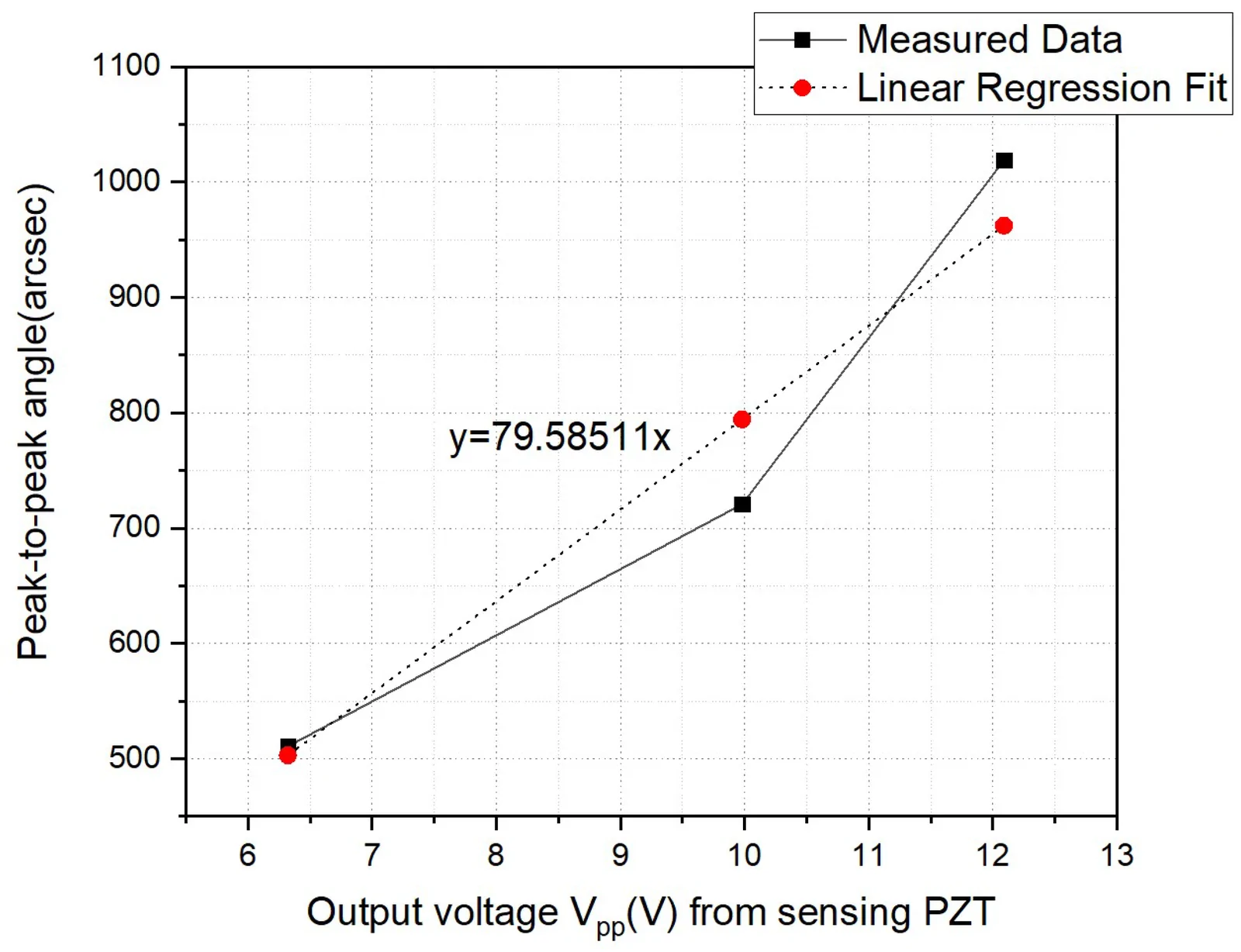
Sensing PZT output voltage vs. peak-to-peak rotation angle amplitude.

To validate the angular velocity amplitude enhancement design presented in Section 4, a cluster dither incorporating the design values in Table 3 was fabricated. In Section 4, the angular velocity amplitude was predicted at the initial design stage using a Q-factor assumed on the basis of the literature. In Section 5, the Q-factor was reevaluated through a frequency-response test of the fabricated prototype, and the angular velocity amplitudes calculated using the reevaluated Q-factor were compared with the test values to assess the ability of the analytical model to reproduce the actual response. Figure 7 shows the test setup used to measure the angular velocity amplitude of the fabricated cluster dither. A sinusoidal signal at the first-mode natural frequency of the cluster dither was generated by a function generator, amplified by a high-voltage amplifier, and applied to the driving PZT attached to the dither spoke. The voltage actually applied to the driving PZT was measured using a Tektronix DPO4104B oscilloscope, and the input voltage to the driving PZT was controlled based on this measured voltage. When a sinusoidal voltage was applied to the driving PZT, the cluster dither underwent sinusoidal motion about its center of rotation. The resulting output voltage of the sensing PZT was measured using an NI-9229 voltage input module installed in an NI cDAQ-9181 and LabVIEW software. To establish the relationship between the sensing PZT output voltage and the rotation-angle amplitude, the sensing PZT output voltage and the ruler displacement were measured under each test condition. A ruler was placed at a distance of R = 35 mm from the center of rotation of the cluster dither, and the vibration—increased by the sinusoidal voltage applied to the driving PZT—was recorded at magnification using a SUNNY SZN45T stereomicroscope. The distance R was set based on the geometry of the finite element model and verified using a digital caliper after the ruler was installed. The two endpoints of the ruler’s range of motion were then identified in the image displayed on the monitor, and the peak-to-peak displacement was measured using the distance calculation function of the image measurement software. Under each test condition, the ruler displacement and the sensing PZT output voltage acquired using the NI cDAQ-9181 and LabVIEW were measured repeatedly several times, and the mean values for each condition were used to determine the relationship between the sensing PZT output voltage and the rotation-angle amplitude. However, because the individual repeated measurements were not retained separately, the standard deviations and confidence intervals associated with repeated measurements could not be calculated. If the average of the measured ruler displacements is ∆L, the rotation-angle amplitude can be calculated from the average displacement as ΔL/R. The measurement results are shown in Figure 8. Figure 8 shows the relationship between the sensing PZT output voltage and the rotation-angle amplitude ΔL/R. Once the rotation-angle amplitude corresponding to the sensing PZT output voltage and the dither driving frequency $\omega_{1}$ are known, the angular velocity of the cluster dither can be calculated using Equation (29).

(29) $$\Omega_{1}=\frac{\Delta L\omega_{1}}{R}$$

As mentioned above, let ΔL_pp_ denote the peak-to-peak displacement of the ruler installed at a distance of R = 35 mm from the center of rotation of the cluster dither. Under the small-angle assumption, the rotation angle shown in Figure 8 is given by Equation (30). Figure 8 shows the relationship between the sensing PZT output voltage and the peak-to-peak rotation-angle amplitude measured at driving PZT applied voltages of 40, 80, and 120 V_pp_. To quantitatively evaluate this relationship, a least-squares linear regression constrained to pass through the origin was performed, and the result is given in Equation (31). Here, θ_pp_ is the peak-to-peak rotation-angle amplitude (arcsec), and V_s_ is the sensing-PZT output voltage (V_pp_). The centered coefficient of determination R^2^ was 0.9335, the residual RMSE was 53.74 arcsec, the residual standard error was 65.82 arcsec, and the maximum absolute residual was 73.48 arcsec. These results indicate an approximately proportional relationship between the sensing PZT output voltage and the rotation-angle amplitude at the three measurement points. However, because the regression has residuals, its estimates cannot be treated as identical to the actual rotation-angle amplitude under each measurement condition. Therefore, the directly measured rotation angles, rather than those estimated from the regression, were used to calculate the angular velocity amplitude in this study. The rotation-angle amplitudes calculated in radians using Equation (30) were converted to arcsec, and the angular velocity amplitudes calculated using Equation (29) were converted to deg/s.

(30) $$\theta_{pp}=\frac{\Delta L_{pp}}{R}$$

(31) $${\hat{\theta }}_{pp}=a\times V_{s}=79.58511\times V_{s}$$

The measurement uncertainty of the rotation-angle amplitude is affected by the uncertainties in the ruler displacement and the distance from the center of rotation. Assuming that the uncertainties in these two measured quantities are independent, the standard uncertainty of the rotation angle is given by Equation (32) [31].

(32) $$u_{\theta }=\theta_{pp}\sqrt{{\left(\frac{u_{\Delta L}}{\Delta L_{pp}}\right)}^{2}+{\left(\frac{u_{R}}{R}\right)}^{2}}$$

In Equation (32), u_ΔL_ is the standard uncertainty of the displacement, and u_R_ is the standard uncertainty of the distance between the center of rotation and the ruler. To evaluate the uncertainty of the ruler displacement measured using the image measurement software, the position-reading resolution at each endpoint was assumed to be 1 μm. Accordingly, the position selection error at each endpoint was modeled as a uniform distribution over ±0.5 μm, yielding a standard uncertainty of 0.4082 μm for the displacement calculated as the difference between the two endpoint positions. The measurement tolerance for the distance between the center of rotation and the ruler was set to ±0.1 mm. Assuming a uniform distribution, the standard uncertainty of this distance was calculated as 0.0577 mm [32]. Applying Equation (32) to the measurement points in Figure 8 yielded standard uncertainties of 2.55–2.93 arcsec and relative standard uncertainties of 0.288–0.499% for the rotation-angle amplitude. For sinusoidal motion, the peak-to-peak angular velocity amplitude is proportional to the rotation-angle amplitude and the resonance frequency. Therefore, applying the law of propagation of uncertainty to Equation (29) gives the standard uncertainty of the angular velocity amplitude as Equation (33) [31]. The uncertainties in the rotation-angle amplitude and the resonance frequency were assumed to be independent.

(33) $$u_{\Omega }=\frac{2\pi }{3600}\sqrt{f^{2}u_{\theta }^{2}+\theta_{pp}^{2}u_{f}^{2}}$$

In Equation (33), f is the experimentally measured resonance frequency, u_f_ is its standard uncertainty, θ_pp_ is the peak-to-peak rotation-angle amplitude directly calculated from the ruler displacement, and u_θ_ is the standard uncertainty of the rotation-angle amplitude. Because the angular velocity amplitude is calculated using the directly measured rotation-angle amplitude and the resonance frequency measured under the corresponding condition, uncertainty was propagated as in Equation (33), assuming that the uncertainties in these two measured quantities were independent. With a frequency measurement tolerance of ±1 Hz and a uniform distribution, the standard uncertainties of the angular velocity amplitude were evaluated as 3.03, 3.21, and 3.56 deg/s at driving-PZT applied voltages of 40, 80, and 120 V_pp_, respectively. The corresponding relative standard uncertainties were 0.506%, 0.382%, and 0.301%. This uncertainty evaluation is based on the specified assumptions for image position readings, distance measurements, and frequency measurements. It does not represent the total measurement uncertainty encompassing the statistical variability of repeated measurements and all error components of the measurement system.

The directly measured peak-to-peak rotation-angle amplitudes shown in Figure 8 at driving-PZT applied voltages of 40, 80, and 120 V_pp_ were 510.7171, 720.7819, and 1018.7922 arcsec, respectively. The resonance frequencies measured under these conditions were 672.0, 668.6, and 665.7 Hz, respectively. Substituting these measured values into Equation (29) yielded peak-to-peak angular velocity amplitudes of 599.0, 841.1, and 1183.7 deg/s, respectively. These results are summarized in Table 5. The regression in Figure 8 was used to evaluate the relationship between the sensing-PZT output voltage and the rotation-angle amplitude and was not used to calculate angular velocity amplitudes. The experimentally identified resonance frequencies ranged from 665.7 to 672.0 Hz. Comparison with the first natural frequency of 670.1 Hz calculated by the finite element analysis in Figure 6 yielded a maximum relative difference of approximately 0.66%, confirming that the finite element model reproduces the actual first resonance frequency of the cluster dither with relatively high accuracy.

**Figure 9:**
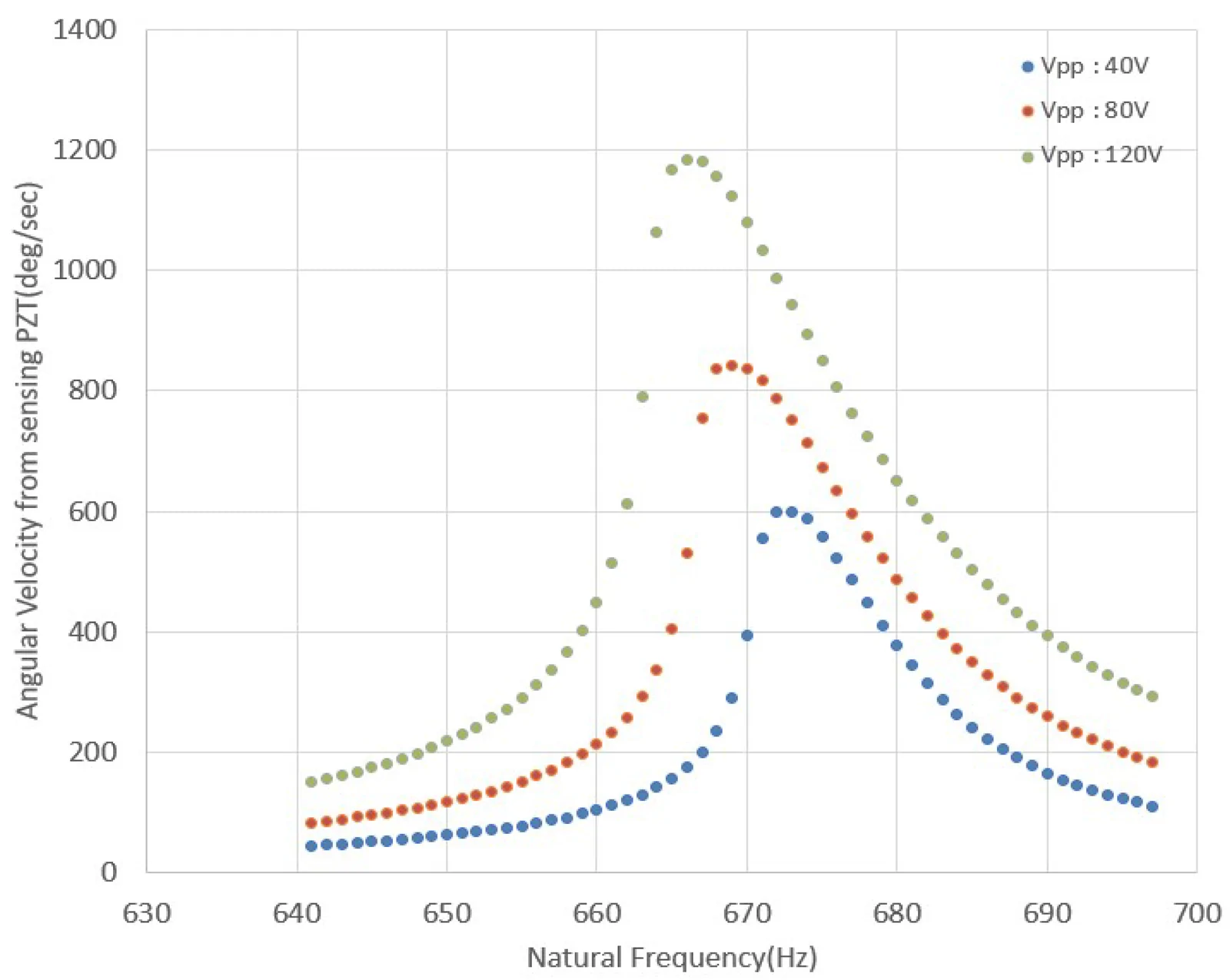
Resonance-frequency and angular velocity amplitude sweep-test results.

In Figure 9, the peak-to-peak angular velocity amplitude reaches its maximum at the resonance frequency, and the maximum value at each applied voltage is summarized in Table 5. First, the measured values in Table 5 were compared with the predicted values in Table 4. Although Table 4 showed a relatively small error at a driving-PZT applied voltage of 40 $V_{pp}$, the prediction errors over the three applied-voltage conditions were 5.4%, 50.1%, and 59.9%, respectively. To identify the cause of the large prediction errors, the appropriateness of assuming an equivalent Q-factor of 100 in Table 4 was examined. To assess this assumption, the Q-factor at each PZT applied voltage was calculated from the frequency-response results in Figure 9 and summarized in Table 6. Because the rates of change in angular velocity amplitude on the left and right sides of the resonance frequency differ substantially, as shown in Figure 9, not only the overall measured average Q-factor, but also $Q_{L}$ and $Q_{R}$, calculated separately for the left and right frequency regions, were obtained. The Q-factors in Table 6 differ considerably depending on the calculation method. This result shows that assuming an equivalent Q-factor of 100 at the initial design stage to predict the angular velocity amplitude for all applied-voltage conditions was not appropriate. In addition, the asymmetry was calculated by substituting $Q_{L}$ and $Q_{R}$ from Table 6 into the Q-factor asymmetry equation in Equation (34), and the results are presented in the last row of Table 6. Left–right asymmetry of the resonance response was observed over the tested driving-voltage range, while the Q-factor asymmetry remained relatively constant in the range of 1.07–1.09. In contrast, the magnitude of the Q-factor varied with driving voltage, indicating that the asymmetry of the resonance response and the voltage dependence of the damping characteristics exhibited different patterns. Meanwhile, the change in the first-mode resonance frequency with applied voltage in Figure 9 was relatively small. Thus, although the change in the first-mode resonance frequency was relatively small over the tested driving-voltage range, the Q-factor and the left–right asymmetry of the resonance response differed with voltage. Changes in resonance frequency alone therefore provide a limited basis for assessing changes in damping characteristics or nonlinear response. In contrast, the variation in Q-factor with applied voltage was relatively larger than the variation in resonance frequency, and a large difference was also observed between $Q_{L}$ and $Q_{R}$, calculated from the left and right sides of the resonance response. The difference between the left- and right-side Q-factors demonstrates the asymmetry of the resonance response. This asymmetry may have been influenced by the nonlinear piezoelectric behavior of the PZT as the applied voltage increased. However, the voltage–strain characteristics, hysteresis, phase response, and temperature dependence of the PZT were not measured independently in this study, so PZT nonlinearity cannot be identified conclusively as the direct cause of these phenomena. Nonlinear PZT behavior can also affect the dynamic characteristics of a structure to which the PZT is attached [33,34]. In addition, asymmetry of the resonance response may arise from the voltage and phase response characteristics of the driving amplifier, the constraints at the dither mounting fixture, or structural nonlinearity. Because these factors were not independently isolated and evaluated in this study, identifying the specific causes of the resonance-response asymmetry remains a subject for future research.

As described above, because the resonance response curve is asymmetric, it is not clear which Q-factor should be used as the equivalent Q-factor for angular velocity amplitude prediction. Therefore, $Q_{L}$, $Q_{R}$, and Q were each substituted into Equation (28) to predict the angular velocity amplitude, and the results are summarized in Table 7. In Table 7, $Q_{R}$ reflects only the damping characteristics of the right-side frequency region of the resonance response and is therefore not suitable for use as an equivalent Q-factor representing the overall resonant angular velocity amplitude. In fact, applying the right-side Q-factor to Equation (28) predicts an angular velocity amplitude substantially lower than the measured value. Table 7 also shows that the angular velocity amplitude of the cluster dither is strongly influenced by the Q-factor and that the Q-factor itself varies with the driving-PZT applied voltage. Comparison of the measured values in Table 5 with the predictions in Table 7 shows that using Q as the equivalent Q-factor produces angular velocity amplitudes most similar to the test results. Therefore, in this study, Q is not defined as a Q-factor that directly represents the physical damping characteristics of the cluster dither; rather, it is selected as an equivalent Q-factor for predicting the angular velocity amplitude in Equation (28) for an asymmetric resonance response. The reason why care is required in selecting an equivalent Q-factor when the Q-factor asymmetry is large can be explained by Equation (27). In a uniformly shaped dither spoke, stress and strain are distributed over a relatively broad region. Therefore, applying the overall measured average Q-factor to the modal integral in Equation (27) is considered to represent the actual damping characteristics reasonably well, keeping the angular velocity amplitude prediction error small.

The angular velocity amplitude prediction errors calculated by comparing the predicted values in Table 7 with the measured values in Table 5 are listed in Table 8. The prediction error ranged from a minimum of 3.3% to a maximum of 4.9%, with a mean of 4.1%. This indicates that the proposed analytical equation reproduces the actual angular velocity amplitudes reasonably well when the equivalent Q-factors measured in resonance-response tests on the same prototype are applied. However, these results do not represent independent a priori prediction accuracy. They also confirm that the variation in Q-factor with operating voltage has an important influence on prediction accuracy. The mean angular velocity amplitude prediction error of 4.1% is comparable to the 5% error under static conditions reported in Reference [4]. However, the two studies differ in their validation conditions, including the structures analyzed and the application of Q-factors. This comparison should therefore be interpreted only as a reference for the level of accuracy of angular velocity amplitude analysis. In addition, the cluster dither fabricated with the angular velocity amplitude enhancement design satisfied the peak-to-peak angular velocity amplitude requirement of 1080 deg/s presented in Section 2 when the driving-PZT applied voltage was 120 $V_{pp}$.

To compare the angular velocity amplitude analysis method proposed in this paper with existing methods, the bending-displacement analysis methods of References [5,7] were used. The angular velocity amplitude equations in References [4,6] are based on the kinetic-energy relationship associated with the tip displacement of a clamped-free cantilever and are therefore difficult to apply directly to the clamped-supported cantilever in this study, whose transverse tip displacement is constrained. In contrast, the methods of References [5,7] can calculate the bending-displacement distribution over the entire beam. In this study, the clamped-supported cantilever boundary conditions were applied to these methods to newly calculate the displacement distribution of the spoke. The displacement distribution was then differentiated along the length to obtain the tip rotation angle, which was converted to angular velocity amplitude assuming sinusoidal motion. The quantitative comparison used the same geometric constraints of the clamped-supported cantilever, PZT excitation moment, frequency for angular velocity conversion, definition of the tip rotation angle, and peak-to-peak amplitude convention. However, the static bending analysis does not account for the steady-state resonant response governed by inertia and damping, whereas the present study considers the resonant dynamic response, including tip rotational inertia and an equivalent Q-factor. The angular velocity amplitude calculated using the existing bending-displacement analysis methods was approximately 10% of the value obtained using the present resonant dynamic analysis. This difference is considered to arise from the different treatment of the static bending response and the steady-state resonant response under the same geometric constraints and excitation conditions. Thus, this comparison does not imply that the existing bending-displacement analysis methods are themselves erroneous. Instead, it shows that evaluating the resonant angular velocity amplitude of a cluster dither requires consideration of the steady-state resonant response and the dynamic amplification associated with damping characteristics.

In an actual cluster dither design, an appropriate applied voltage should be selected within the range that satisfies the required angular velocity amplitude. The maximum design applied voltage of the cluster dither fabricated in this study was set to 160 $V_{pp}$. In the test, the dither angular velocity amplitude was measured at applied voltages of 40, 80, and 120 $V_{pp}$. At 120 $V_{pp}$, the measured peak-to-peak angular velocity amplitude was 1183.7 deg/s, which sufficiently satisfied the required value of 1080 deg/s. This corresponds to a peak angular velocity amplitude of 591.85 deg/s, approximately 9.6% above the target value of 540 deg/s set in this study. Therefore, in an actual design, an appropriate applied voltage can be selected that satisfies the required angular velocity amplitude without exceeding the maximum design applied voltage of 160 $V_{pp}$.

(34) $$A_{Q}=\frac{2\left|Q_{L}-Q_{R}\right|}{Q_{L}+Q_{R}}$$

To evaluate the effect of Q-factor selection on the angular velocity calculation, Table 9 compares the initial assumed value Q = 100, a Q-factor measured at 40 V_pp_ and applied at other voltages, and Q-factors determined separately at each voltage. With a fixed Q = 100, the mean prediction error across the three voltage conditions was approximately 38.5%. Applying Q = 91.8, identified at 40 V_pp_, to all voltages produced an overall mean error of 29.7%; the cross-application mean error at 80 and 120 V_pp_, which were not used for Q-factor identification, was 42.9%. Applying voltage-specific measured Q-factors reduced the mean calculation error to approximately 4.1%. The results demonstrate the strong influence of voltage-dependent Q-factors on predicted angular velocity amplitude. In particular, a Q-factor measured at one voltage may lead to large errors when applied at others, so voltage-dependent damping should be considered for accurate prediction. Results obtained using voltage-specific measured Q-factors are conditional calculations incorporating experimentally determined damping; they are not independent a priori predictions.

In addition, Table 10 summarizes the cluster-dither rotation-angle amplitudes for different driving-PZT voltages. Sinusoidal voltages of 40, 80, and 120 V_pp_ were applied, and the peak-to-peak sensing-PZT output voltages and ruler displacements were measured. The measured ruler displacements and the distance R = 35 mm from the rotation center yielded directly calculated rotation-angle amplitudes of 510.72, 720.78, and 1018.79 arcsec, respectively. These results are direct measurements under the individual test conditions, rather than estimates from the regression in Figure 8, and describe the change in mechanical rotational response with driving voltage.

## 6. Conclusions

In this paper, an angular velocity amplitude equation was derived from the equation of motion while considering the clamped-supported cantilever boundary conditions of the cluster dither and the inertial effect of the end attachment, and the principal design variables affecting angular velocity amplitude were analyzed. Based on the analysis results, methods for increasing angular velocity amplitude were proposed, including selection of the optimum PZT attachment position based on the first-mode shape function, reduction in the effective end mass, and application of a material with a high Q-factor. A cluster dither incorporating the proposed methods was also designed and fabricated, and test evaluations were performed.

To verify the proposed angular velocity amplitude analysis and enhancement design method, the cluster dither was designed as follows. SUS304 was used as the dither material, and the dither spokes were designed with a uniform geometry. The PZT attachment start position was set to 0.6*L* so that the difference in mode-shape slopes over the PZT attachment region was maximized, and the effective mass at the dither end was reduced. M&S and test evaluations were performed on the cluster dither incorporating the angular velocity amplitude enhancement design. Several candidate Q-factors obtained from the Q-factor measurement test were each applied to the angular velocity amplitude prediction and compared with the test results to select the equivalent Q-factor most suitable for angular velocity amplitude prediction. Comparison between predictions based on the selected equivalent Q-factor and the measured angular velocities yielded a mean angular velocity amplitude prediction error of 4.1%. In addition, when the PZT applied voltage was 120 $V_{pp}$, the peak-to-peak angular velocity amplitude of the dither was 1183.7 deg/s, exceeding the requirement of 1080 deg/s by approximately 9.6%. Based on the mean prediction error of 4.1% and satisfaction of the required angular velocity amplitude, the applicability of the proposed cluster dither angular velocity amplitude analysis and enhancement method was confirmed. However, satisfying the 1080 deg/s requirement verifies the dynamic performance of the cluster dither itself and does not directly measure improvements in the actual RLG angle random walk or zero-signal stability. Because angle random walk, zero-signal stability, power consumption, mass and dimensions, and parasitic vibration characteristics were not separately measured against a conventional dither, quantitative comparison of these characteristics will require future integrated testing.

In future work, the fabricated ring laser gyroscopes will be mounted on the cluster dither, and the angular velocity will be measured at the inertial-measurement-unit level with natural-frequency control applied. Through these IMU-level angular velocity measurement tests, the proposed cluster dither angular velocity amplitude analysis and enhancement method will be further validated. In addition, the present study analyzed angular velocity amplitude based on the resonance response of the first bending mode and an equivalent Q-factor. Therefore, additional nonlinear modeling will be required under conditions in which higher-order modes have a significant influence or strong PZT nonlinearity occurs under high-voltage drive.

## Figures and Tables

**Table 1:** Comparison of previous methods and the proposed cluster dither angular velocity amplitude analysis method.

Comparison Item	References [4,6]	References [5,7]	Present Study
Subject of analysis	Single-axis RLG mechanical dither	Composite-layer spoke of a single-axis RLG mechanical dither	Three-axis RLG cluster dither
Analysis method	Static bending deformation and energy-equivalence method	Moment equilibrium and strain compatibility	Equation-of-motion-based modal analysis
Boundary conditions of original study	Clamped-free cantilever	Clamped-free cantilever	Clamped-supported cantilever
PZT effects	Bending moment induced by piezoelectric deformation	Composite bending stiffness of PZT and adhesive layers; piezoelectric deformation	Sinusoidal excitation bending moment generated by the PZT
Inertial effects	Considered through the energy relation	Inertial forces neglected in the transverse-displacement calculation	Effective mass and end inertia included
Treatment of damping	Q-factor measured, but excluded from the amplitude equation	Equivalent damping ratio presented, but excluded from the displacement equation	Equivalent Q-factor included in the resonant amplitude equation
Principal inputs	Beam geometry and properties; PZT moment; displacement- and energy-related variables	Composite-layer geometry and properties; piezoelectric strain and flexural rigidity	Beam geometry and properties; mode shape; PZT moment; end inertia; equivalent Q-factor
Principal calculated result	Maximum rotation angle and angular velocity amplitude	Transverse-displacement distribution along the beam	Resonant angular velocity amplitude
Validation	Finite-element analysis and angular velocity amplitude test	Transverse-displacement test	Angular velocity amplitude test; mean error 4.1%
Angular velocity amplitude calculation	Direct calculation using energy equivalence based on end displacement	Not performed in original study; can be converted from the beam displacement distribution to end rotation and angular velocity amplitude	Direct calculation of steady-state resonant angular velocity amplitude based on the equation of motion
Conditions of applicability	Deformation and kinetic-energy relation of a clamped-free cantilever	Evaluation of beam displacement distribution under a static bending moment	Sinusoidal excitation and resonance-response characteristics of a clamped-supported cantilever
Applicability to present structure	Direct use of the existing end-displacement-based angular velocity equation is difficult	Requires recalculation of displacement using clamped-supported boundary conditions and conversion to end rotation and angular velocity amplitude	Direct resonant angular velocity analysis including end rotational inertia and damping

**Table 2:** Effects of cluster dither design variables on angular velocity amplitude.

No.	Related Variable	Cluster Dither Design Variable	Effect on Angular Velocity Amplitude
1	Equation (28)	PZT bending moment ($M_{eq}$)	∝ $M_{eq}$
2	Equation (28)	Shape-function slope ($Y_{1}^{\prime }\left(L\right)$, $Y_{1}^{\prime }\left(x_{c}\right)-Y_{1}^{\prime }\left(x_{f}\right)$)	∝ $Y_{1}^{\prime }\left(L\right)$, $Y_{1}^{\prime }\left(x_{c}\right)-Y_{1}^{\prime }\left(x_{f}\right)$
3	Equation (28)	Cluster dither natural frequency ($\omega_{1}$)	Contains a ∝ 1/$\omega_{1}$ term, but interpretation of its effect in isolation is limited
4	Equation (28)	Effective mass ($m_{r}$)	∝ 1/$m_{r}$
5	Equation (28)	Dither-spoke Q-factor (Q=1/2ζ)	∝ Q
6	$\omega_{1}$	Dither-spoke stiffness ($E_{i}I_{i}$)	∝ 1/($E_{i}I_{i}$)^1/2^
7	$\omega_{1}$	Dither-spoke area (ρA)	∝ 1/(ρA)^1/2^
8	$m_{r}$	Dither-spoke area (ρA)	∝ (ρA)
9	$\omega_{1}$	Dither-spoke depth ($h_{b}$)	∝ 1/($h_{b}$)^1/2^
10	$I_{i}$		∝ $h_{b}$
11	$m_{r}$		∝ $h_{b}$
12	$\omega_{1}$	Dither-spoke thickness ($t_{b}$)	∝ 1/($t_{b}$)^3/2^
13	$I_{i}$		∝ ($t_{b}$)^3^
14	$m_{r}$		∝ $t_{b}$
15	$\omega_{1}$	End mass ($m_{e}$)	∝ 1/($m_{e}$)^1/2^
16	$m_{r}$		∝ $m_{e}$
17	$\omega_{1}$	End moment of inertia ($J_{e}$)	∝ 1/($J_{e}$)^1/2^
18	$m_{r}$		∝ $J_{e}$

**Table 3:** Cluster dither design-variable values.

Cluster Dither Design Variable	Design Value
PZT attachment start point ($x_{c}$)	14.54 mm (0.6*L*)
PZT attachment end point ($x_{f}$)	24.24 mm
Representative natural frequency applied to the angular velocity amplitude analysis ($\omega_{1}$)	670 Hz
Typical loss factor of dither material	0.00095~0.0013
Dither-spoke depth ($h_{b}$)	19.0 mm
Dither-spoke length (*L*)	24.24 mm
Dither-spoke thickness ($t_{b}$)	1.80 mm
End moment of inertia ($J_{e}+m_{e}d^{2}$)	4.19 × 10^−5^ kg·m^2^
PZT dimensions (mm) ($L_{0}$ × $h_{b}$ × $t_{p}$)	9.6 × 19 × 0.3 mm

**Table 4:** Predicted peak-to-peak angular velocity amplitudes of the cluster dither.

Driving PZT Input Voltage (V_pp_)	Predicted Peak-to-Peak Angular Velocity Amplitude
40	631.13
80	1262.26
120	1893.39

**Table 5:** Measured peak-to-peak angular velocity amplitudes of the cluster dither.

Input Voltage (V_pp_)	Measured Peak-to-Peak Angular Velocity Amplitude (deg/s)
40	599.0
80	841.1
120	1183.7

**Table 6:** Measured Q-factors of the cluster dither.

	Input Voltage (V_pp_)	40	80	120
Q-Factor				
$Q_{L}$		197.7	152.6	128.0
$Q_{R}$		59.8	44.8	39.0
Q		91.8	69.9	59.8
$A_{Q}$		1.07	1.09	1.07

**Table 7:** Peak-to-peak angular velocity amplitudes predicted using Equation (28).

	Input Voltage (V_pp_)	40		80		120	
Q-Factor							
$Q_{L}$		1247.7	deg/s	1926.2	deg/s	2423.5	deg/s
$Q_{R}$		377.4		565.5		738.4	
Q		579.4		882.3		1132.2	

**Table 8:** Angular velocity amplitude prediction errors.

Input Voltage (V_pp_)	Angular Velocity Amplitude Prediction Error
40	3.3%
80	4.9%
120	4.3%
Average	4.1%

**Table 9:** Comparison of angular velocity amplitude predictions and errors.

Q-Factor Selection Method	Comparison Item	40 V_pp_	80 V_pp_	120 V_pp_
Measured values	Angular velocity amplitude (deg/s)	599.0	841.1	1183.7
Initial design assumption	Applied Q-factor	100	100	100
	Predicted angular velocity amplitude (deg/s)	631.13	1262.26	1893.39
	Prediction error (%)	5.4	50.1	60.0
Reuse of the Q-factor identified at 40 V_pp_	Applied Q-factor	91.8	91.8	91.8
	Predicted angular velocity amplitude (deg/s)	579.4	1158.8	1738.1
	Prediction error (%)	3.3	39.0	46.8
Voltage-specific Q-factors measured from resonance responses	Applied Q-factor	91.8	69.9	59.8
	Predicted angular velocity amplitude (deg/s)	579.4	882.3	1132.2
	Prediction error (%)	3.3	4.9	4.3

**Table 10:** Rotation-angle amplitude results for different driving-PZT voltages.

Driving-PZT Input Voltage (V_pp_)	Sensing-PZT Output Voltage (V_pp_)	Measured Rotation-Angle Amplitude (Arcsec, Peak-to-Peak)
40	6.32	510.72
80	9.98	720.78
120	12.09	1018.79

## Data Availability

Dataset available on request from the authors.

## Abbreviations

The following abbreviations are used in this manuscript:

IMU	Inertial Measurement Unit
M&S	Modeling and Simulation
RLG	Ring Laser Gyroscope
PZT	Lead Zirconate Titanate
ARW	Angle Random Walk
